# Heated Allergens and Induction of Tolerance in Food Allergic Children

**DOI:** 10.3390/nu5062028

**Published:** 2013-06-05

**Authors:** Merryn Netting, Maria Makrides, Michael Gold, Patrick Quinn, Irmeli Penttila

**Affiliations:** 1 Women’s and Children’s Health Research Institute, University of Adelaide, 72 King William Road, North Adelaide, SA 5006, Australia; E-Mails: merryn.netting@adelaide.edu.au (M.N.); maria.makrides@adelaide.edu.au (M.M.); 2 Children’s Youth and Women’s Health Network, University of Adelaide, 72 King William Road, North Adelaide, SA 5006, Australia; E-Mails: michael.gold@adelaide.edu.au (M.G.); patrick.quinn@adelaide.edu.au (P.Q.)

**Keywords:** egg, milk, allergy, heated allergens, tolerance, oral, immunotherapy

## Abstract

Food allergies are one of the first manifestations of allergic disease and have been shown to significantly impact on general health perception, parental emotional distress and family activities. It is estimated that in the Western world, almost one in ten children have an IgE-mediated allergy. Cow’s milk and egg allergy are common childhood allergies. Until recently, children with food allergy were advised to avoid all dietary exposure to the allergen to which they were sensitive, in the thought that consumption would exacerbate their allergy. However, recent publications indicate that up to 70% of children with egg allergy can tolerate egg baked in a cake or muffin without apparent reaction. Likewise, up to 75% of children can tolerate baked goods containing cow’s milk, and these children demonstrate IgE and IgG4 profiles indicative of tolerance development. This article will review the current literature regarding the use of heated food allergens as immunotherapy for children with cow’s milk and egg allergy.

## 1. Introduction

Food allergies have become increasingly common around the world, and it is estimated that globally, about 220–520 million people may suffer from food allergy [1]. Although it is possible to develop an IgE-mediated allergy to any food, most individuals with allergies react to one or a combination of nine common foods: cow’s milk, soy, egg, wheat, peanut, tree nuts, sesame, fish and shellfish [2]. 

Food allergies are one of the first manifestations of allergic disease and have been shown to significantly impact on general health perception, parental emotional distress and family activities [3]. Young children, in particular, are at risk of developing food allergy, and it is estimated that 5% to 8% of toddlers are affected, compared with 1% to 2% of adults [1]. The incidence of allergic diseases is increasing globally, and this poses a major burden to healthcare costs in every country around the world [1].

Many children outgrow their food allergy; however, recent data suggest that the natural history of food allergy has changed, and it is now common for allergies to persist to late childhood and early adulthood [4,5].

In an affected patient with a food allergy, management consists of removal of the food from the diet and provision of an action plan for management of any accidental exposure to the food protein. More recently, complete exclusion of the food from a food allergic individual has been questioned. It is possible that including the allergenic food in the diet has potential to increase the likelihood of a child outgrowing the allergy more quickly or to allow a certain amount of the food to be tolerated [6]. Complete and absolute avoidance of a food allergen may not be necessary in a child’s diet if they show tolerance to that food in an alternative format. Many children with cow’s milk or egg allergies tolerate foods containing baked milk [7] or egg protein [8], and the hypothesis that this might be associated with a move towards tolerance of the protein has moved dietary management strategies away from strict avoidance to allowing the inclusion of the baked protein in the diet if it is tolerated [9,10,11]. 

With the rising incidence in food allergies there has been interest in improving their management, with the aim of offering a treatment strategy that does not solely rely on avoidance of food proteins in the diet. Immunotherapy is used successfully for managing venom and pollen allergies, and thus, there has been increasing research into the use of immunotherapy to manage common food allergies [12]. This review will summarize current research related to oral immunotherapy for management of IgE-mediated cow’s milk and egg allergy and, in particular, the potential for heated proteins to be used as vehicles for oral immunotherapy.

## 2. Development of Oral Tolerance

The intestine is the largest immune organ in the body, and it works to protect the body from a constant onslaught of pathogens and ingested proteins derived from the outside environment by secreting Immunoglobulin A (IgA) and activating immune cells to fight pathogens or to act as regulatory cells to maintain gut homeostasis. Gut-associated lymphoid tissue (GALT), in particular, Peyer’s Patches, are involved in the induction of immunity and maintenance of gut homeostasis [13,14]. The gut epithelium, along with dendritic cells, plays an active role in this process by continually sampling the gut luminal contents [15]. The interactions between microbes, epithelium and GALT are involved in developing memory of the immune system and are essential for the development of oral tolerance [16].

Our understanding of the exact mechanisms involved in the development of tolerance to an allergen is evolving; however, the precise mechanism(s) by which the normal intestinal immune system responds to food and its involvement in the development of allergy remains essentially unresolved [17,18]. Development of oral tolerance is dependent on a number of early events, including allergen exposure (age, dose and timing), gut colonisation and the influence of maternal milk [19,20,21,22,23,24,25]. The resulting regulated immune response is an antigen driven process involving repeated exposure to the antigen and establishment of healthy gut colonization with commensal bacteria [25].

Development of oral tolerance to a food allergen involves early changes at the level of the gut mucosa [26]. Initially, there is a need to establish a local immunosuppressive gut milieu dominated by immunomodulatory cytokines, such as IL-10 and TGFβ, which control inflammation, non-specifically [27]. This environment is then conducive to the development of a highly regulated systemic response and differentiation of antigen-specific (CD4^+^CD25^+^FOXP3^+^) regulatory T-cells (T-regs), essential for immune homeostasis in the intestine [23,24,28,29,30,31]. However, the role of these regulatory T-cells in food allergic disease and regulation of immune response development to allergens in the gut remains unclear. Studies have shown that transcription factor Foxp3 is critical for the development and function of T-regs. A variant of IPEX syndrome, which is characterised by autoimmune and severe allergic symptoms, including severe enteropathy, food allergies, atopic dermatitis, hyper-IgE and eosinophilia, is associated with a mutation within an upstream non-coding region of Foxp3 that affects its function [32]. Recent studies have also shown that in the intestine retinoic acid producing dendritic cells and Foxp3^+^ T-regs interact to induce oral tolerance [33,34]. With regard to food allergy, the frequency of Foxp3^+^ cells in the periphery is low in individuals with food allergies, and children with food allergy have reduced T-regulatory cell function [35]. Other studies have shown that a higher frequency of food allergen-specific T-regulatory cells correlates with a milder clinical phenotype and favourable prognosis [36]. 

## 3. Immunotherapy for Food Allergies

Given the potential of exposure to a small amount of allergen to promote tolerance to that allergen and the successful use of immunotherapy for management of venom and pollen allergies, there has been increasing research into the use of immunotherapy to manage common food allergies [12]. Early use of subcutaneous immunotherapy for food allergy resulted in severe reactions, and so, recent focus has been on oral immunotherapy [37]. Forms of oral immunotherapy to treat food allergy include specific oral tolerance induction (SOTI) [12], where the allergen is ingested, or sublingual immunotherapy (SLIT), where a minute amount (picograms) of allergen, in the form of allergen extract or diluted allergen, is placed under the tongue [12,38]. Both approaches start with a ‘build up phase’, initially starting with very small, diluted doses of the food protein with the amount of protein included in the dose slowly increased in a stepwise manner. This is followed by a “maintenance phase” with a consistent dose of allergen given at regular intervals. The regimes are followed with a “discontinuation phase” and a “tolerance food challenge” to test the effectiveness of the immunotherapy treatment. The entire process may take 12 months or longer to complete. Adverse events are common during oral immunotherapy—this risk of an adverse event increases with illness, exercise, menses and every time the dose of the food is increased [39,40]. If regular consumption of the allergen is discontinued during the maintenance phase, there is a risk that tolerance will be broken, and an allergic reaction may follow exposure [38]. For food allergy, the endpoint of immunotherapy may not always be tolerance—the therapy may result in *desensitization*, an increase in the antigen dose required to elicit symptoms arising from a transient altered immune response and dependent on regular (daily) exposure to the allergen, or the therapy may result in true *tolerance* [41]—the ability to ingest the allergen without any symptoms once regular exposure has ceased, owing to persistent changes in the immune response. Desensitization still has a benefit in that larger doses of the allergen can be tolerated before eliciting a hypersensitivity reaction, thus lessening the potential for an anaphylactic reaction from accidental exposure. 

Oral immunotherapy has been attempted for many food proteins, but we have specifically chosen to discuss cow’s milk and egg oral immunotherapy, because of the interest in using baked forms of these foods as a treatment strategy. There is one Cochrane review from 2012 [42] on oral immunotherapy for cow’s milk allergy, but none for egg. The Cochrane review included 16 studies, representing five trials and concluded that milk oral immunotherapy can lead to desensitization in the majority of individuals, although development of long term-tolerance has not been established. 

Key trials reporting cow’s milk oral immunotherapy and egg oral immunotherapy in the last 10 years are summarized in Table 1, Table 2. Nine studies reported outcomes of cow’s milk oral immunotherapy and also nine for egg oral immunotherapy. These include three randomized controlled trials (RCTs) for cow’s milk SOTI [38,43,44] and three RCTs investigating egg oral immunotherapy [45,46,47], but most of the studies report on open, uncontrolled prospective trials. We have added one trial [38] that was not included in the cows’ milk immunotherapy Cochrane review. These studies have limitations. The studies utilized highly variable selection criteria, dosing regimens and maintenance doses, making them difficult to compare. The individual studies reported have limited statistical power, as only a small number of children were included in the treatment group.

All of the 18 studies reported SOTI, and one study reported results of a SLIT/SOTI combination protocol for cow’s milk immunotherapy. Keet *et al*. [38] compared the outcome of children with cow’s milk allergy who were given a SLIT protocol initially and then randomized to a low or high dose SOTI protocol or SLIT alone. More children in the SLIT followed by the high dose SOTI group passed the final oral food challenge after six weeks compared to those in SLIT alone or the lower SOTI dose.

Dosing regimens varied from 15 mL to 200 mL cow’s milk and 0.3 g to 60 g of egg for the maintenance phase. Furthermore the allergen used for the reported regimes varied—for example fresh raw whole egg, fresh raw egg white, pasteurized egg white, pasteurized whole egg, lightly cooked egg have all been used for the egg oral immunotherapy studies. Most of the cow’s milk immunotherapy studies used fresh cow’s milk, but some reported use of dried non fat milk powder. Some of the studies asked participants to consume the maintenance dose on a daily basis, and some required the dose to be consumed every 2–3 days.

**Table 1:** Summary of key egg-specific oral immunotherapy trials.

Study	Study Design	Subjects *N*, age (range) in active groups	Protocol	Clinical Outcomes	Clinical Results: if no endpoint challenge, tolerance was determined by tolerance of maintenance dose	Immunological Outcomes
**Burks, 2012** [46]	RCT	*N* = 40 7 years (5–11)	SOTI Egg white powder *vs.* corn starch Blinded until 10 month challenge	*Maintenance*: up to 2 g egg white powder daily (1/3 egg) *Endpoint challenge*: OFC at 10 and 22 months (5 g EW powder) If child passed 22 month OFC, OIT ceased and egg-free diet for 4–6 weeks. At 24 months, these children had an OFC with 10 g egg white powder and one cooked egg. Evaluations at 30 and 36 months per telephone.	**At 10 month OFC:** 22/35 tolerant 1/35 withdrew **At 22 month OFC** 30/34 tolerant **At 24 months OFC:** 11/29 tolerant **30 months phone call:** 11/11 tolerant **36 months phone call:** 10/10 tolerant	SPT ↓ IgE ↓ IgG ↑ Egg-specific basophil activation ↓
**Meglio 2013** [45]	RCT	*N* = 10, median age 8.4 years	SOTI Adjunct Therapy Raw fresh egg *vs.* egg-free diet	*Maintenance*: 25 mL raw egg three-times/week, alternatively could consume foods corresponding to about three eggs per week. *Endpoint challenge*: No	**At six months:** 8/10 tolerant 1/10 partially tolerant 1/10 withdrew (2/10 in control group achieved tolerance of raw egg in same time period.)	SPT ↓ SPT ↓ IgG4 ovomucoid ↔ IgG4 ovalbumin ↑ Cytokines and transforming growth factors (IL-5 ↑)
**Dello Iacono 2013** [47]	RCT	*N* = 10 7 years, 7 months (5–11)	SOTI Raw fresh egg *vs.* egg free diet	*Maintenance:* 40 mL raw egg (one small egg). *Endpoint challenge*: No	**At six months:** 9/10 partially tolerant 1/10 no tolerance	SPT ↓ IgE ↓
**Patriarca, *et al*. 2007** [48]	Prospective, open study with reference group	*N* = 14 3–16 years	Sublingual initially, then higher doses orally. Adjunct therapy Raw fresh egg	*Maintenance*: one egg 2–3 times/week. *Endpoint challenge*: No	10/14 tolerant 1/14 partially tolerant 1/14 failed 2/14 withdrew	SPT ↓ IgE ↓ IgG4 ↑
**Fuentes-Aparico 2012** [49]	Prospective, open study with reference group	*N* = 19 mean 9.2 years (4–14)	SOTI Pasteurised egg powder	*Maintenance*: 10 g powdered egg (equivalent to one egg) *Endpoint challenge*: No Follow up at six and 12 months	16/19 tolerant 2/19 withdrew 1/19 partially tolerant to cooked egg	IgE ↔ IgG4 ↑ T-cell subtypes: B-cells, NK cells, NKT cells, CD8+T-cells: ↔ Effector memory CD4+T-cells (TEM): ↓ CD4+ recent thymic emigrants (RTEs): ↑ CD38/RO neg T-cells: ↑ Cytokines: IL-12, IL4, IL-13 & IL-β: ↔ Th1 cytokines (IL-2, TNF-α and IFNγ): ↓ Th2 cytokines ( IL-5 and IL-10) & Th9 (IL-9), Th22 (IL-22) and Th17 cytokines (IL-17A): ↓ IL-13 ↑(trend NS)
**Patriarca *et al*. 2003** [50]	Prospective, open study	*N* = 12 <16 years	SOTI Adjunct therapy Raw fresh egg	*Maintenance*: one egg (50 mL) 2-3 times/week. 3-8 months *Endpoint challenge*: No	10/12 tolerant 2/12 withdrew	SPT ↓ ↔ IgE ↓ IgG4 ↑
**Buchanan *et al*. 2007** [51]	Prospective, open study	*N* = 7, 4 years (14–84 months)	SOTI Egg white powder	*Maintenance*: 300 mg daily for 24 months. *Endpoint challenge*: DBPCFC at 24 months (10 g egg white). If passed, followed by open scrambled egg challenge.	**24 month DBPCFC** 4/7 tolerant. 3/7 failed, but tolerated more egg	IgE ↓ (NS) IgG4 ↑
**Itoh, 2010** [52]	Prospective, open study	*N* = 6, mean 9.7 years (7–12)	SOTI Raw powdered egg white and lightly cooked (whole) egg	*Maintenance*: one whole egg (60 g) At least two times/week. *Endpoint challenge*: OFC to powdered egg at 9–12 months after starting maintenance.	**9–12 months OFC:** 3/6 tolerant 3/6 failed At 16 months: 6/6 tolerated >60 g heated egg.	SPT ↔ IgE ↑ then ↓ IgG4 ↑ Histamine release test ↓; Th1/Th2 ratio ↓ at six months, not at 12 months; IL-4, IFNγ ↔; IL-10 ↓, TGFβ1 ↑
**Garcia Rodriguez, 2011** [53] **Urra, 2012** [54]	Prospective, open study	*N* = 23 mean 8.1 years (5–17)	SOTI Pasteurised raw egg white and whole cooked egg	*Maintenance*: one cooked egg daily for three months. Exposure then extended to once in 48 h and, at six months once, in 72 h. *Endpoint challenge*: No	20/23 tolerant 1/23 withdrew 2/23 switched to slow protocol and achieved tolerance.	SPT ↓ IgE ↓ IgG4 ↑ CD4 + FoxP3 + cells: ↑

N, number; RCT, randomized controlled trial; SOTI, specific oral tolerance induction; OFC, Oral Food Challenge; DBPCFC, Double Blind, Placebo Controlled Food Challenge; EPSPT, Endpoint titration skin prick testing; SPT, skin prick test; NS, not significant.

**Table 2:** Summary of key cow’s milk specific oral immunotherapy trials.

Study and Study Type	Study Design	Subjects *N*, age (range) in active groups	Protocol	Outcomes	Clinical Results: if no endpoint challenge, tolerance was determined by tolerance of maintenance dose.	Immunological Outcomes
**Skripak 2008** [43]	RCT	*N* = 13 9 years (6–21)	SOTI Dried powdered milk *vs.* Profree	*Maintenance*: 500 mg milk protein (15 mL milk) *Endpoint challenge*: DBPCFC up to 2000 mg, cumulative dose of 8 g milk protein	12/13 tolerant 1/13 withdrew	IgE ↑ IgG4 ↑
**Martorell 2011** [44]	RCT	*N* = 30 * 2 years (2–3)	SOTI Fresh cow’s milk *vs.* milk-free diet	*Maintenance*: 200 mL daily plus unlimited dairy products. *Endpoint challenge*: only in two children who did not tolerate SOTI.	**At 16 weeks:** 27/30 tolerant 1/30 partially tolerant 1/30—no tolerance 1/30—withdrew	IgE ↓
**Keet 2012** [38]	RCT	*N* = 30 6–16 years SLIT group *n* = 10 median age: 8 year (6–11), OITB group *n* = 10 9 year (6–15), OITA group *n* = 10 8 years (6–16)	SLIT alone, *vs.* SLIT followed by SOTI (high or low dose). SLIT, target dose 7 mg. OITA, target dose of 2 g. OITB, target dose of 1 g. SLIT, CM allergenic extract SOTI, dried non-fat milk powder	*Maintenance*: SLIT, target dose 7 mg. OITA = target dose of 2 g. OITB = target dose of 1 g. *Endpoint challenge*: OFC to 8 g cow’s milk protein at 32 and 80 weeks (on therapy) *and* at 81 and 86 weeks (off therapy)	28/30 completed protocol 2/30 withdrew **Overall at end of protocol** SLIT/SLIT: 1/10 tolerant SLIT/OITB: 3/10 tolerant SLIT/OITA: 5/10 tolerant **Challenge threshold at 32 weeks (12 weeks of maintenance)** SLIT: 7× increase OITB: 64× increase OITA: 79× increase **Desensitization *vs.* tolerance:** **81 week challenge (one week off therapy):** 2/10 (SLIT/OITB group) failed, both requiring adrenalin. **86 week challenge:** SLIT/OITB group 1/10 failed SLIT/OITA group 3/10 failed	EPSPT IgE ↓ (in OIT, but not SLIT) IgG4 ↑ basophil histamine release ↓ (NS), constitutive CD63 expression ↓ (SLIT/SLIT group), CD203c expression ↓, intracellular spleen tyrosine kinase (Syk) levels ↔
**Patriarca 2003** [50]	Prospective, open study with reference group	*N* = 16 <16 years	SOTI adjunct therapy fresh cow’s milk	*Maintenance*: 120 mL milk at least 2–3 times/week *Endpoint challenge*: No	10/16 tolerant 3/16 interrupted schedule 3/16 withdrew	SPT ↓ ↔ IgE ↓ IgG4 ↑
**Patriarca 2007** [48]	Prospective, open study with reference group	*N* = 14 3–16 years	SLIT, then SOTI. adjunct therapy fresh cow’s milk	*Maintenance*: 130 mL milk at least 2–3 times a week *Endpoint challenge*: No	10/14 tolerant 1/14 partially tolerant 1/14 failed 2/14 withdrew	SPT ↓ ↔ IgE ↓ IgG4 ↑
**Meglio 2004** [55] **& 2008** [56]	Prospective, open study	*N* = 21 6 years (5–10)	SOTI Adjunct Therapy Fresh cow’s milk	*Maintenance*: 200 mL milk per day for six months *Endpoint challenge*: No	15/21 tolerant (8/15 asymptomatic 7/15 symptomatic and managed with adjunct therapy) 3/21 withdrew **After four years** 13/20 tolerant 1/20 partially tolerant	SPT EPST ↓ IgE ↔ Faecal blood: no occult blood reported.
**Narisety 2009** [57] Follow up of [43]	Prospective, open study	*N* = 15 6–16 years, tolerating 75 mL cow’s milk.	Tolerant children increased dose of dried powdered milk by 50% every two weeks.	*Maintenance*: median dose 7000 mg milk protein (≈200 mL milk) *Endpoint challenge*: OFC at 13–75 weeks cumulative milk dose of 16 mg milk protein (480 mL)	**At 13–75 weeks** 6/16 tolerant 7/16 partially tolerant 2/16 ongoing symptoms with maintenance, so no OFC	EPSPT ↓ IgE ↓ IgG4 ↑
**Alvaro 2012** [58]	Prospective, open study	*N* = 66 mean 8 years (44/66 with anaphylaxis to cow’s milk )	SOTI fresh cow’s milk	*Maintenance*: 200 mL daily *Endpoint challenge*: No	**non anaphylactic group:** 16/22 tolerant 6/22 partially tolerant **anaphylactic group:** 35/44 tolerant. 7/44 partially tolerant 1/44 tolerant to 1 mL 1/44 withdrew	IgE ↓
**Bedoret 2012** [59]	Prospective, open study	*N* = 10 8years (7–17)	SOTI adjunct therapy fresh cow’s milk	*Maintenance*: 2000 mg cow’s milk protein (60 mL) *Endpoint challenge*: DBPCFC at week 24 Cumulative dose of 7250 mg, then open challenge of 120–240 mL milk.	**At week 24** 9/10 tolerant 1/10 withdrew	SPT ↓ IgE ↓ IgG4 ↑ CD4 T-cell proliferation ↓ T-reg cells ↔ IFNγ /IL-4 ratio ↑ Basophil activation ↓

SLIT, sublingual immunotherapy.

Not all of the studies assessed actual tolerance to cow’s milk or egg at the end of the intervention. Some used a double blinded, placebo controlled food challenge, the recognized “gold standard” for diagnosis of food allergy and some of the trials used open oral food challenges. The end point challenges varied in terms of the type of food used for challenge, with some testing the raw cow’s milk or egg and some testing cooked egg for the case of egg immunotherapy. The volume of cow’s milk or egg given in the final challenge varied. 

Many children who underwent immune therapy were able to reach tolerance to cow’s milk or egg in these studies however overall there was a high incidence of adverse reactions and a risk of loss of clinical desensitization after small periods off therapy [38]. Keet *et al.* reported loss of tolerance in as little as one week off SOTI [38]. The longest follow up period after the oral immunotherapy reported was three years for egg allergy [46] and four years for cow’s milk allergy [56]. There are no safety data available testing the efficacy of oral immunotherapy beyond this length of time. 

Many of the human intervention studies investigating clinical outcomes of individuals undergoing oral immunotherapy to cow’s milk and egg have used skin prick testing as a marker of IgE levels or have measured serum specific IgE / IgG4 levels against the specific allergens to look for changes in the immune system towards tolerance (Table 1, Table 2). Only the more recent studies have begun to investigate changes at the cellular level [38,46,49,53]. Production of cytokines (IL10, TGFß and IFNγ) by T-cells influences the production of protective antibody responses by B cells including secretion of allergen-specific IgG4 and IgA and later inhibition of IgE. Immunotherapy has also been shown to lead to increases in protective IgG (1 and 4) and IgA, a decrease in IgE and a shift in the Th1/Th2 balance towards Th1, along with a decrease in T-cell proliferation and cytokine responses to allergens. Importantly there are increases in regulatory T-cells along with an increased production of IL-10 and TGFß implying that oral tolerance is being established [41]. 

Oral immunotherapy to egg and cow’s milk shows potential as a management strategy for children with cow’s milk and egg allergies and is able to induce desensitization and promote immune changes indicative of moves towards tolerance. However, SOTI carries a risk of adverse events both during the initiation phase and after periods off therapy, and the ideal treatment protocol is still to be identified. For researchers, a strategy that would facilitate this is the development of harmonized protocols, or, at least, harmonization of reporting of studies into SOTI to enable reporting, so that they can be clearly compared.

## 4. Use of Baked Proteins for Oral Immunotherapy—An Alternative Approach?

The form in which an oral allergen is delivered can influence the development of tolerance and the subsequent immune response profile generated. The studies summarized in Table 1, Table 2 all used raw proteins for the initial immunotherapy. If tolerance is achieved, in an individual, to raw uncooked proteins, then other forms of the food will also be tolerated. This has probably been the reason why centres have chosen to use raw foods as the vehicle for SOTI in the above reported clinical trials. Liquid or powders are also easy to measure, making the doses easy to deliver. However, SOTI using raw proteins is associated with a high rate of adverse events and often does not lead to permanent tolerance.

It is recognized that some individuals with cow’s milk and egg allergies are able to tolerate the proteins they are allergic to in the form of baked goods. Traditionally, allergic individuals were advised to avoid all forms of the allergenic protein in their diet, even if the individual was including the allergen in cooked food, for example, without any adverse events, in the belief that this could delay the resolution of the allergy [6]. However, it is now thought that achievement of tolerance to a heat treated protein may be the first step towards “outgrowing” the allergy and inducing tolerance [10]. In a longitudinal study of British children [60], tolerance to well-cooked egg (baked in a sponge cake) was gained at a median of 67 months (5.7 years) compared to 127 months (10.6 years) for raw egg. Resolution still continued to occur to cooked egg up to the age of 158 months (13.2 years) and for raw egg up to 182 months (15.2 years). There are no similar longitudinal studies that assess the natural history of resolution to products containing baked milk proteins; however, Wood *et al*. [61] recently published data on a multicentre cohort of children with cow’s milk allergy. Of 293 participants, the median age of resolution of cow’s milk allergy was 63 months (5.25 years). Of the 155 children with unresolved allergy, 32 (20.6%) were able to tolerate products containing baked milk at the five year time point.

## 5. Why Are Baked Proteins Different?

Inclusion of baked milk or baked egg in the diet enables many foods that were previously avoided to be included in the diet, and this liberalization results in an improved quality of life for the child with food allergies. It is possible that regular inclusion of products containing the baked protein reduces the risk of reactions, due to accidental exposure to products containing egg or milk. 

Proteins are three-dimensional molecules held together by electrostatic charge. IgE binding sites (or epitopes) may be sequential (several amino acids in a row) or conformational (part of the shape of the protein) [62]. Differences in allergenicity are, in part, due to changes in the structure of the proteins when heated, which affect the specific conformational IgE binding sites on the protein molecule. It is recognised that heat treatment of a protein can result in conformational changes in epitopes by affecting hydrogen bonds within the protein and, thus, affecting the ability of the IgE molecule to bind [63]. In the case of egg, heat treatment destroys the conformational epitopes that some individuals form IgE against, thus allowing ingestion of the egg without any adverse reaction. Some proteins are more susceptible to heat treatment than others, for example, the egg protein ovalbumin is more susceptible to heat treatment than ovomucoid [64]. In addition to altering the epitopes, heating the egg protein with wheat (for example, in a cake) forms a food matrix, also acting to reduce the allergenicity of the protein by affecting the digestibility of the proteins or making the IgE binding sites less accessible [64,65,66]. Other means of altering the allergenicity of a food protein include hydrolysis, acidification and other forms of food processing [67].

## 6. Studies Assessing Tolerance to Baked Proteins in Individuals with Egg Allergy and Cow’s Milk Allergy

In 2008, Konstantinou [9] described the retrospective evaluation of 94 children (median age 24 months range 12–48 months) with egg allergy (*n* = 55) or IgE sensitization to egg (*n* = 39). Ninety percent of the children tolerated an open oral food challenge to baked egg (containing 1.5 g baked egg protein). The 87 children who tolerated baked egg were then allowed to freely consume baked egg in their diet for six months and, then, were given a whole egg challenge. Only four out of 87 (4.6%) children reacted to this challenge.

Lemon Mule *et al*. [8] were the first to study immune changes associated with consumption of baked egg in a wheat matrix by individuals with egg allergy. They enrolled 117 children with diagnosed egg allergy (mean age 6.6, range 1.6–18.6 years) and challenged them with heated (baked) egg in the form of a muffin or a waffle—70% of the study group tolerated the baked egg and were then advised to include baked egg in their diet. The group was followed up at three, six and 12 months. Regular consumption of heated egg was associated with decreasing skin prick test (SPT) weal sizes to egg white and increasing ovalbumin- and ovomucoid-specific IgG4 levels. Intestinal permeability assessed by measurement of urinary clearance of non-metabolized sugars was no different between children consuming baked egg and those not consuming baked egg, and the children continued to grow well. 

The study group was followed for six years, and the results of subsequent food challenges were reported by Nowak-Wegrzyn [68] and Leonard [69]. After incorporating heated egg into their diet, 58% of children eventually tolerated regular egg (French toast or scrambled egg) in their diets after a median of 16.6 months (interquartile range (IQR) 12.9–37.1 months). Children in the treatment group (consuming baked egg) were 14.6-times more likely to develop regular egg tolerance than children in the retrospective comparison group, and the children consuming baked egg developed tolerance earlier (median 50.0 *vs.* 78.7 months; *p* < 0.0001) compared with those who did not.

Nowak-Wegrzyn *et al*. [70] challenged 100 cow’s milk allergic children (average age 7.5 years, range 2.1–17.3 years) with cow’s milk baked in waffles or muffins. Seventy-five percent of the cow’s milk allergic children tolerated baked milk. Those who tolerated baked milk were asked to consume products containing baked milk at home for three months and, then, were re-evaluated. Immune response (serum-specific IgE and IgG4), growth and intestinal permeability were also monitored. After three months, children consuming baked milk products had significantly smaller SPT and higher casein—IgG4 compared with baseline. On repeat challenges with uncooked cow’s milk, the group of children who tolerated heated cow’s milk outgrew their milk allergy quicker than the group who did not tolerate heated cow’s milk in their diet [7]. 

Kim [7] reported the results of the long-term follow-up of the group initially described by Nowak-Wegrzyn. The initial baked milk challenge was tolerated by 65 of 88 children. Compared with the children strictly avoiding milk, baked milk-tolerant children were more likely to become tolerant to unheated milk than baked milk-reactive children (Odds Ratio 2.8 (95% Confidence Interval, 4.8–162.7); *p* < 0.01). No difference was noted in the milk-specific IgE levels between groups; however, both the casein IgE and β-lactoglobulin IgE values in the baked milk tolerant group decreased significantly over time. There was a significant increase in the median casein IgG4 value, but not the β-lactoglobulin IgG4 level from base line to final visit in the baked milk tolerant group. Eosinophilic esophagitis, which has been identified as a potential complication of SOTI [51], was reported in the baked milk study, but not the baked egg study. Two subjects in the active group and five in the comparison group had eosinophilic esophagitis, but this was not related to the intervention.

The baked egg and milk studies by Konstantinou, Lemon Mule *et al*. and Nowak-Wegrzyn *et al*. [8,9,70] are promising, but need substantiation, as the studies were not randomized controlled trials, and the results may be due to comparisons of different phenotypes of milk or egg allergy, changes with time or other unidentified confounders. Neither of the studies tested tolerance to the raw protein after a period of avoidance of the baked products, so it is unknown whether the effect was permanent.

There are several advantages of using baked products as a mode of inducing tolerance to egg or cow’s milk protein. Once tolerance to a baked product is proven, the child is able to include it in their diet on a regular basis. Compared with traditional SOTI protocols, this means that there is less cost in terms of hospital admission times and inconvenience to the family, as there is no intense “up dosing” phase required. This approach is potentially safer compared with protocols, which rely on parents making up and administering the appropriate diluted dose of the allergen at home. The ability to consume baked products also moves the child towards a more normal, less restricted diet.

At present, there are no tests that predict tolerance to baked egg or baked milk proteins. Foods containing baked egg are reported to elicit anaphylaxis in egg allergic individuals, and as such, individuals chosen for this therapy should be carefully screened [9]. 

Furthermore, to date, there are few studies reporting immune mechanisms after oral immunotherapy, and this is one area that requires further research to help guide development of immunotherapy protocols. There is one study by Shreffler *et al*. that has shown a significantly higher percentage of proliferating casein-specific CD25/CD271 T-cells (regulatory T-cells involved in tolerance induction) from casein-induced peripheral blood mononuclear cell cultures taken from extensively heated milk-tolerant children compared with cells from extensively heated milk-reactive children. They showed no significant difference between the groups in the frequency of polyclonal T-cells or casein-specific effector T-cells. However, casein-specific regulatory T-cells correlated with the phenotype of having a mild transient milk allergy and favourable prognosis [36]. Induction of these regulatory T-cells is critical for the development or oral tolerance, and therefore, the study supports the concept of using heated allergens for tolerance induction. 

While allergen specific oral immunotherapy has potential, there are significant questions regarding effectiveness and safety [71]. We have focused our review on tolerance induction to egg and milk; however, peanut allergy is also a significant health issue. Unfortunately, using heated peanut as a potential immunotherapeutic may not be feasible, as some forms of heat treatment (roasting) enables exposure of new epitopes to form, rendering this form of peanut more allergenic than boiled or fried peanut [72].

## 7. Animal Studies Supporting the Use of Heated Proteins as Immunotherapeutic Agents

There has been some work in animal models of allergy describing the effect on ingestion of heated egg proteins, but little about heated milk proteins. Martos [64] demonstrated that heat treatment of ovalbumin and ovomucoid reduces the allergenicity partly by changing the digestibility of the protein. Egg-sensitized C3H/HeJ mice were challenged with either untreated or heated ovalbumin or ovomucoid. Oral challenge with the unheated protein induced anaphylaxis in all of the mice, but not when challenged with the heated proteins. Even when challenged intraperitoneally, the majority of the mice only displayed mild reactions. In a separate experiment, mice fed heated ovalbumin had minimal T-cell proliferation in the mesenteric lymph nodes, or Peyer’s Patches, compared with those consuming unheated ovalbumin, suggesting that heating ovalbumin affects the intestinal absorption of the intact form capable of triggering effector cells and T-cells. In this same paper, Martos [64] also reported that heated ovalbumin and ovomucoid still retained the ability to bind to serum from egg allergic children on immunoblotting, indicating that the epitopes were still intact. 

Leonard [73] used a mouse model of SOTI to show that heated egg protein (ovomucoid) is able to desensitize egg allergic mice as effectively as unheated egg protein. C3H/HeJ mice sensitized to egg were treated with increasing doses of egg white or heated or native ovomucoid. Mice were challenged with ovalbumin or ovomucoid either one day or two weeks after the end of the desensitization period. At day 1, significantly fewer of the SOTI treated mice experienced anaphylaxis when compared with controls; however, when the mice were re-challenged at two weeks after ceasing SOTI, the previously desensitized mice were no longer protected from anaphylaxis. Mice given heated ovomucoid were completely protected against anaphylaxis compared with controls, as were mice given a higher dose egg white treatment compared with a low dose treatment. Antigen specific IL-13, IL-10 and IFNγ responses were all significantly lower in SOTI treated mice compared with controls, and this difference remained two weeks post treatment. Whole genome microarray analysis of jejunum of sensitized control mice compared with those who were treated with SOTI indicated that administration of SOTI may lead to the regulation of a subset of genes in the intestine, including some expressed by intestinal epithelial cells. They postulated that the reduction in both the IL-10 and IL-13 after the SOTI indicated that the effect was related to an overall suppression rather than a shift in TH1/Th2 response.

As discussed, human studies investigating the ingestion of heated cow’s milk and heated egg in allergic individuals demonstrated that this was associated with decreased skin test weal diameters and allergen-specific IgE levels and increased allergen-specific IgG4 levels [8,74]. Further studies are required to further quantify the immune changes that occur with ingestion of heated proteins to enable us to understand if ingestion of heated egg or heated cow’s milk affects the natural history of egg and cow’s milk allergies when compared with strict avoidance. Other important information that is required includes identification of diagnostic tests to accurately predict reactions to heat treated proteins and target potential candidates for this form of therapy, development of optimal protocols and evaluation of their efficacy and safety.

## 8. Conclusions

Oral induction of tolerance is a promising option for the management of food allergy, particularly with the increasing numbers of children being diagnosed with food allergies worldwide. However, SOTI with raw proteins is not free of risks, and there is much work to be done before it can be safely recommended as routine. The inclusion of baked cow’s milk and egg proteins in the diet of children with IgE-mediated cow’s milk and egg allergies appears to move the children towards a more tolerant immune profile, and the use of baked proteins is appealing, as it is safe, improves quality of life as the child moves towards a more normal diet and mimics the natural history of development of tolerance.

Areas for future research include unravelling the underlying mechanisms in the development of tolerance to a food. This will aid in the understanding and identification of the optimal route and protocols for dosing during the desensitization phase and also help to identify markers showing tolerance development as opposed to desensitization. Diagnostic tests to correctly identify candidates to approach regarding SOTI are required as are steps taken to optimize the safety of protocols. Harmonized study protocols or at least harmonization of reporting of studies into SOTI to enable reporting, so that they can be compared, is essential to the progression of this important area of research. Longer-term trials comparing children of like age groups and allergy phenotypes are required. Quality of life data should also be gathered from the individuals undergoing SOTI, as this has been largely absent from the studies reported, so far. Much more research is required to investigate if this will help children outgrow their allergies quicker, thus lessening the burden of managing food allergic disease.
